# Number of siblings, birth order, and childhood overweight: a population-based cross-sectional study in Japan

**DOI:** 10.1186/1471-2458-12-766

**Published:** 2012-09-11

**Authors:** Hirotaka Ochiai, Takako Shirasawa, Tadahiro Ohtsu, Rimei Nishimura, Aya Morimoto, Ritsuko Obuchi, Hiromi Hoshino, Naoko Tajima, Akatsuki Kokaze

**Affiliations:** 1 Department of Public Health, Showa University School of Medicine, 1-5-8 Hatanodai, Shinagawa-ku, 142-8555, Tokyo, Japan; 2 Division of Diabetes, Metabolism and Endocrinology, Department of Internal Medicine, Jikei University School of Medicine, 3-25-8 Nishi-Shinbashi Minato-ku, Tokyo, 105-8461, Japan; 3, Jikei University School of Medicine, 3-25-8 Nishi-Shinbashi Minato-ku, Tokyo, 105-8461, Japan

**Keywords:** Sibling, Birth-order, Childhood overweight, Public health

## Abstract

**Background:**

Although several studies have investigated the relationship between the number of siblings or birth order and childhood overweight, the results are inconsistent. In addition, little is known about the impact of having older or younger siblings on overweight among elementary schoolchildren. The present population-based study investigated the relationship of the number of siblings and birth order with childhood overweight and evaluated the impact of having younger or older siblings on childhood overweight among elementary schoolchildren in Japan.

**Methods:**

Subjects comprised fourth-grade schoolchildren (age, 9–10 years) in Ina Town during 1999–2009. Information about subjects’ sex, age, birth weight, birth order, number of siblings, lifestyle, and parents’ age, height, and weight was collected by a self-administered questionnaire, while measurements of subjects’ height and weight were done at school. Childhood overweight was defined according to age- and sex-specific cut-off points proposed by the International Obesity Task Force. A logistic regression model was used to calculate the odds ratio (OR) and 95% confidence intervals (95% CI) of "number of siblings" or "birth order" for overweight.

**Results:**

Data from 4026 children were analyzed. Only children (OR: 2.13, 95% CI: 1.45-3.14) and youngest children (1.56, 1.13-2.16) significantly increased ORs for overweight compared with middle children. A larger number of siblings decreased the OR for overweight (*P* for trend < 0.001). Although there was no statistically significant relationship between a larger number of older siblings and overweight, a larger number of younger siblings resulted in a lower OR for overweight (*P* for trend < 0.001).

**Conclusions:**

Being an only or youngest child was associated with childhood overweight, and having a larger number of younger siblings was negatively associated with overweight. The present study suggests that public health interventions to prevent childhood overweight need to focus on children from these family backgrounds.

## Background

Childhood obesity has adverse effects on the cardiovascular system and the negative effects of obesity can persist, which predicts a strong link between childhood obesity and morbidity/mortality in adulthood [1]. Persistent obesity has also been reported to be associated with poorer employment and relationship outcomes in a cohort study [2]. Moreover, obese children are more likely to experience psychological or psychiatric problems than non-obese children [1]. Therefore, obesity among children is a serious public health problem that can affect their entire life.

Adolescence is a critical period for the development of obesity that persists into adulthood [3]. A previous study showed that because obesity is difficult to treat, public health efforts need to be directed toward prevention [4]. Therefore, it is important to be able to identify individuals at high risk for overweight/obesity among preadolescent children and to reinforce appropriate interventions for the prevention of overweight/obesity before adolescence.

Childhood overweight or obesity has been shown to be associated with family variables including parental age and obesity, the number of siblings, and socioeconomic status [5-16]. In fact, these variables have been reported to be the most important variables associated with childhood obesity [17]. Among family variables, several studies have investigated the relationship between siblings and childhood overweight or obesity [11-16]. However, the results of these studies are inconsistent; some have reported that the number of siblings was associated with overweight or obesity [11-14], whereas others have shown no association between the number of siblings and childhood overweight or obesity [15,16]. In addition, little is known about the impact of having older or younger siblings on overweight among elementary schoolchildren. Therefore, it is important to examine the impact of the presence and number of siblings on childhood overweight among schoolchildren in order to help identify individuals at high risk for overweight. We hypothesized that only child was associated with overweight, a larger number of siblings resulted in a lower risk of overweight, and the impact of having younger siblings on overweight was different from that of having older siblings on overweight.

Accordingly, the aim of the present study was to investigate the relationship of the number of siblings and birth order with childhood overweight and to evaluate the impact of having younger or older siblings on childhood overweight among elementary schoolchildren in Japan.

## Methods

In addition to annual national health checkups performed in accordance with the School Health Law, Ina Town, located in Saitama Prefecture, Japan, has implemented a unique health-promotion program, consisting of blood and physical examinations for fourth- and seventh-graders, since 1994. The purpose has been the prevention and improvement of obesity among schoolchildren in Ina Town. Details of this health promotion program are available elsewhere [18-21]. The present study was conducted as a part of that program.

### Study subjects

Subjects comprised all fourth-grade schoolchildren aged 9 or 10 years in Ina Town from 1999 to 2009 (N = 4106). Informed consent to participate in the study was obtained from each child’s parent or guardian. This study was approved by the Medical Ethics Committee of Showa University School of Medicine.

Of 4106 subjects, 25 refused to participate in the study (participation rate: 99.4%) and 55 were excluded in the analysis due to incomplete data. Thus, a total of 4026 children were analyzed.

### Questionnaire survey

A self-administered questionnaire was distributed to each subject by the school teacher in the elementary school. Then, each subject and the parent or guardian completed the questionnaire. The questionnaire was returned to the school teacher.

The following information was collected by a self-administered questionnaire completed by each child: sex, age, exercise outside of physical education class (daily, sometimes, or none), and eating speed (fast, medium, or slow).

In addition, each subject’s parent or guardian was asked to complete a self-administered questionnaire about his or her child’s birth weight, the frequency of eating breakfast per week (daily, sometimes, or none), bedtime, wake-up time, and family information (number of siblings, the subject’s birth order, and the parents’ age, height, and weight). The frequency of eating breakfast per week was categorized into two group; skipping breakfast (sometimes or none) and not skipping breakfast (daily). Sleeping hours were calculated based on the bedtime and wake-up time.

### Anthropometric measurements

Anthropometric measurements were performed in a standardized manner. Each subject’s height and weight was measured in the school’s infirmary or in a designated room that protected privacy during the procedures. Subjects were lightly clothed and barefoot. Height was measured to the nearest 0.1 cm using a stadiometer and body weight was measured with a scale to the nearest 0.1 kg. Body mass index (BMI) was calculated as weight (kg) divided by height (m) squared. All information was recorded annually from 1999 to 2009.

### Definition of overweight

Overweight in childhood was defined according to the age- and sex-specific cut-off points proposed by the International Obesity Task Force [22]. Parental overweight was defined as a BMI ≥ 25 kg/m^2^ according to the criteria of the World Health Organization [23].

### Data analysis

The number of siblings was categorized into three groups: none (only child), one, or two or more. Birth order was categorized into four groups: only child, oldest child, youngest child, or middle child. Rather than being classified as either youngest or oldest, only children were placed in the "only child" category in this study. The middle child category included all children who had "one or more younger sibling(s)" and "one or more older sibling(s)". The Kolmogorov-Smirnov test was used to test the normality of distribution. The chi-square or the Wilcoxon rank-sum test was used to compare various characteristics between the overweight group and the non-overweight group. A logistic regression model was used to calculate the odds ratio (OR) and 95% confidence interval (95% CI) of "number of siblings" or "birth order" for overweight, and then potential confounders were adjusted. Variables that have been reported to be related to childhood overweight [5-7,24] and that were different between the overweight and the non-overweight group with a *P* value < 0.1 were considered as potential confounders. For adjustment, sex, birth weight, eating speed, exercise, sleeping hours, mother's age, paternal overweight, and maternal overweight were put in the model. A *P* value < 0.05 was considered statistically significant and a value of 0.05 ≤ *P* < 0.1 was regarded as marginally significant. All statistical analyses were performed using Statistical Analysis System (SAS, version 9.2).

## Results

Table 1 shows the characteristics of the non-overweight and overweight groups among study participants. The proportion of boys was significantly higher in the overweight than in the non-overweight group. There was a significant difference between the non-overweight and the overweight group in birth weight. Children who did not exercise and who were fast eaters were frequently found in the overweight group. A marginally significant difference between the overweight and non-overweight groups was observed regarding sleeping hours.

**Table 1:** Characteristics of the non-overweight and overweight groups

**Variables**	**Non-overweight (N = 3370) n(%)**	**Overweight (N = 656) n(%)**	***P*value**^**a**^
Sex			
Boys	1705 (50.6)	382 (58.2)	<0.001
Girls	1665 (49.4)	274 (41.8)	
Age (years)	9.0 (9.36)^b^ (n = 3370)	9.0 (9.37)^b^ (n = 656)	0.607
Birth weight (g)			
<2500	348 (10.3)	53 (8.1)	<0.001
2500-2999	1140 (33.8)	180 (27.4)	
3000-3499	1417 (42.1)	289 (44.1)	
3500-3999	427 (12.7)	114 (17.4)	
4000+	38 (1.1)	20 (3.1)	
Exercise			
None	654 (19.9)	153 (24.1)	<0.001
Sometimes	902 (27.5)	199 (31.3)	
Daily	1725 (52.6)	283 (44.6)	
Skipping breakfast			
Yes	120 (3.6)	19 (2.9)	0.390
No	3239 (96.4)	636 (97.1)	
Eating speed			
Fast	481 (14.4)	215 (33.5)	<0.001
Medium	1903 (57.1)	356 (55.5)	
Slow	947 (28.4)	71 (11.1)	
Sleeping hours			
10.0+	491 (15.2)	83 (13.3)	0.075
9.0-9.9	1963 (60.7)	363 (58.1)	
8.0-8.9	709 (21.9)	160 (25.6)	
<8.0	69 (2.1)	19 (3.0)	

^a^Chi-squared test or Wilcoxon rank-sum test.

^b^Values are median (mean).

Family characteristics of the non-overweight and overweight groups are shown in Table 2. Children with paternal overweight or maternal overweight were more frequently found in overweight group than in the non-overweight group. Significant differences between the non-overweight and the overweight group were observed regarding mother’s age, the number of siblings, and birth order.

**Table 2:** Family characteristics of the non-overweight and overweight groups

**Variables**	**Non-overweight (N = 3370) n(%)**	**Overweight (N = 656) n(%)**	***P*value**^**a**^
Father’s age (years)			
<30	18 (0.6)	2 (0.3)	0.172
30-39	1337 (42.3)	229 (37.9)	
40-49	1637 (51.8)	337 (55.7)	
50+	169 (5.4)	37 (6.1)	
Paternal overweight			
Yes	758 (22.5)	246 (37.5)	<0.001
No	2612 (77.5)	410 (62.5)	
Mother’s age (years)			
<30	44 (1.3)	9 (1.4)	0.013
30-39	2022 (61.6)	379 (60.1)	
40-49	1192 (36.3)	229 (36.3)	
50+	26 (0.8)	14 (2.2)	
Maternal overweight			
Yes	269 (8.0)	140 (21.3)	<0.001
No	3101 (92.0)	516 (78.7)	
Number of siblings			
0 (only child)	328 (9.7)	95 (14.5)	<0.001
1	1855 (55.0)	360 (54.9)	
2 or more	1187 (35.2)	201 (30.6)	
Birth order			
Only child	328 (9.7)	95 (14.5)	<0.001
Oldest child	1387 (41.2)	230 (35.1)	
Youngest child	1216 (36.1)	261 (39.8)	
Middle child	439 (13.0)	70 (10.7)	

^a^Chi-squared test.

Crude and adjusted ORs of the number of siblings and birth order for overweight were calculated (Table 3). A larger number of siblings brought about a lower OR for overweight (*P* for trend < 0.001). Being an only child (OR: 2.13, 95% CI: 1.45-3.14) or youngest child (1.56, 1.13-2.16) significantly increased the OR for overweight compared with being a middle child. Even when oldest, youngest, and middle children were combined into one category (children with siblings) and that category was used as a reference variable, being an only child significantly increased the OR for being overweight (1.76, 1.32-2.33). These results were similar for boys and girls (data not shown). The interaction of sex and the number of siblings, birth order, or being an only child on overweight was not statistically significant.

**Table 3:** Crude and adjusted odds ratios of number of siblings and birth order for overweight

**Variables**	**Total N**	**Overweight n (%)**	**Crude**		**Adjusted**^**a**^	
			**OR**	**95% CI**	**OR**	**95% CI**
Number of siblings (%)						
0 (only child)	423	95 (22.5)	1.00		1.00	
1	2215	360 (16.3)	0.67	0.52-0.86	0.62	0.46-0.83
2 or more	1388	201 (14.5)	0.59	0.45-0.77	0.50	0.36-0.68
			*P* for trend < 0.001		*P* for trend < 0.001	
Birth order (%)						
Only child	423	95 (22.5)	1.82	1.29-2.55	2.13	1.45-3.14
Oldest child	1617	230 (14.2)	1.04	0.78-1.39	1.01	0.73-1.40
Youngest child	1477	261 (17.7)	1.35	1.01-1.79	1.56	1.13-2.16
Middle child	509	70 (13.8)	1.00		1.00	

OR; odds ratio; CI: confidence interval.

^a^Adjusted for sex, birth weight, eating speed, exercise, sleeping hours, mother’s age, paternal overweight, and maternal overweight.

Next, the adjusted ORs of the number of older or younger siblings for overweight were evaluated (Table 4). A larger number of younger siblings resulted in a lower OR for overweight (*P* for trend < 0.001). However, there was no significant relationship between the number of older siblings and overweight. Even if these analyses were limited to each sex, results were similar between boys and girls (data not shown). No interaction of sex and the number of older or younger siblings on overweight was observed.

**Table 4:** Adjusted odds ratios of number of younger/older siblings for overweight

**Variables**	**Total N**	**Overweight n (%)**	**AOR (95% CI)**
Number of younger siblings			
0 (Only child)	423	95 (22.5)	1.00
1 (Oldest child)	1190	173 (14.5)	0.50 (0.36-0.70)
2 or more (Oldest child)	427	57 (13.4)	0.33 (0.21-0.51)
			*P* for trend <0.001
Number of older siblings			
0 (Only child)	423	95 (22.5)	1.00
1 (Youngest child)	1025	187 (18.2)	0.73 (0.53-1.01)
2 or more (Youngest child)	452	74 (16.4)	0.70 (0.47-1.05)
			*P* for trend = 0.078

AOR: adjusted odds ratio, CI: confidence interval.

Adjusted for sex, birth weight, eating speed, exercise, sleeping hours, mother’s age, paternal overweight, and maternal overweight.

## Discussion

### Baseline characteristics of study participants

In the present study, some baseline characteristics differed between the overweight and the non-overweight groups; for example, children who did not exercise and fast eaters were more frequently found in the overweight group. Similarly, children with a lower number of sleeping hours were marginally significantly more likely to be in the overweight group. Moreover, some family characteristics, such as paternal overweight, maternal overweight, and mother’s age, were also significantly different between the non-overweight and overweight groups. For instance, parental overweight was more frequently observed among overweight group. These results are consistent with other study results; some studies have reported that lifestyle factors such as exercise, eating speed, and sleeping hours were associated with childhood overweight [5,25,26], while others have shown that parental overweight was a risk factor for overweight or obesity [6,27]. Therefore, we adjusted for these factors in the analysis to evaluate the relationship of the number of siblings and birth order with overweight.

### Siblings and overweight

As shown in Table 3, a larger number of siblings resulted in a lower OR for overweight. A recent study reported that a small number of siblings was a risk factor for obesity [12]. Furthermore, children with no siblings were reported to be at a higher risk for overweight or obesity and number of siblings is a possible risk factor for the development of overweight and obesity [11,13]. These previous studies are consistent with our findings.

One explanation for these results is that additional sibling(s) might serve as a stimulus for child-to-child interactions, cooperative play, or activities that increase the time each child devotes to physical activity [11]. A previous study suggested that siblings was related to physical activity [28]. However, even when physical activity level (exercise) was adjusted in the analysis to evaluate the relationship between the number of siblings and overweight in the present study, the relationship remained. Therefore, factors other than exercise could be associated with the relationship between sibling and overweight.

Another explanation could be due to the relationship between the number of sibling and the amount of food per one child; for instance, a previous study showed that the amount of food for each child in large families is smaller than that in small families [29]. In fact, one variable associated with increased nutritional risk was reported to be having more than one sibling [30]. Moreover, a previous study showed that only children had significantly higher intakes of many nutrients and nutrients/1000 kcal than children with sibling(s) [31], which might be due to the fact that a mother with an only child is more concerned with persuading her child to eat and grow than is a mother with several children [29]. Therefore, additional siblings may decrease the availability of food for each child, resulting in the reduction of the OR for overweight.

In our study, a statistically significant trend was observed between the number of younger siblings and overweight. However, the number of older siblings did not result in a significantly decreased OR for overweight (Table 4). Older siblings are reported to serve as role models or share the caretaking role with parents [11]. Therefore, having one or more older siblings may not decrease the availability of food for each child. As a result, the number of older siblings might not bring about an increased nutritional risk. Because information on total energy intake was not obtained in the present study, further study is necessary to verify our results.

### Birth order and overweight

In the present study, only and youngest children showed significantly increased ORs for overweight. Being an only child has been reported to be associated with a higher risk for overweight or obesity [8,11,13], which is consistent with our findings. In contrast, some studies have reported that oldest children were significantly associated with overweight or increased adiposity [32,33], which is not consistent with the results of this study. The reason for these discrepancies may be the difference in definition of the “oldest child” status. Only children were included in the oldest children (first-born status) category in previous studies [32,33], whereas only children were not considered “oldest children” in the present study.

In addition, the increase in OR of only children was more pronounced than that of youngest children in our study (Table 3). These results could be due to the association between a larger number of siblings, especially a larger number of younger siblings, and decreased ORs for overweight in the present study.

The present results suggest that being an only child or youngest child are risk factors for overweight. Because these statuses are non-modifiable factors, appropriate intervention for the prevention of childhood overweight should focus on these at-risk children.

### Limitations

In the present study, we did not obtain information regarding grandparent(s), exposure to environmental tobacco smoking, and socio-economic status such as family income, which have been reported to be associated with childhood overweight [34-37]. The amount of food for each child in the family could be influenced by grandparent(s) and socio-economic status of subjects, which might affect our study results. In fact, a recent study reported that socioeconomic position was associated with the number of children in Japan [38]. Therefore, the possibility of residual confounding is not deniable. However, the influence of socioeconomic status in Japan could be smaller than that in the other countries because social inequalities in Japan are less expressed than those in the other countries [39]. Furthermore, the number of siblings, specifically two or more siblings, was reported to be associated with risk of nutrition, independent of socio-economic status [30].

Another limitation is that birth interval was not considered. For example, the impact of a 19-year old sibling on the physique (overweight or non-overweight) of a 9-year old subject (10 years difference) could be different from that of a 10-year old sibling on the physique of a 9-year old subject (1 year difference). Furthermore, if a youngest child became a middle child due to the birth of a new sibling just a few weeks before the information was collected in our study, the middle child status might not be substantially associated with his or her present physique. Therefore, birth interval should be considered to verify our study results in the future.

Thirdly, more objective measurements about physical activity and eating speed could be needed because they were self-reported as general items. In previous studies, study subjects were asked how many days in a typical week they were physically active for 60 min or more [40], while physical activity was measured with actigraph [41]. Additionally, eating speed was measured by laboratory-based methods in a past study [42]. Although it would have been better if we could have used the objective measurements, it could be difficult to apply such methods to a large epidemiological study such as this study. However, in future study, it will be necessary to conduct a questionnaire survey for collecting more detailed information about physical activity and to use a validated questionnaire for obtaining eating speed because the validation of the questionnaire of eating speed in our study was not confirmed.

Finally, subjects in the present study were from one town of Japan. Therefore, it might be difficult to generalize the present study results to other population.

## Conclusions

Being an only or youngest child was associated with childhood overweight. Furthermore, a larger number of siblings, specifically younger siblings, was negatively associated with overweight. These results suggest that public health interventions to prevent childhood overweight should focus on these at-risk children.

## Abbreviations

BMI: Body mass index; OR: Odds ratio; CI: Confidence interval.

## Competing interests

The authors declare that they have no competing interests.

## Authors’ contributions

HO, TS, and TO planned the present study. RN, AM, and HH contributed to improving the study in a meaningful way. HO drafted the manuscript. TS, RN, and AM performed the data collection. HH supported the data collection. TS was in charge of the supervision of the data collection. HO, TO, RO, and AK contributed to the statistical analysis. NT and AK made substantial contributions to the conception of this study and the revision of the manuscript. All authors read and approved the final manuscript.

## Pre-publication history

The pre-publication history for this paper can be accessed here:

http://www.biomedcentral.com/1471-2458/12/766/prepub
